# Radiomic Signatures Derived from Diffusion-Weighted Imaging for the Assessment of Breast Cancer Receptor Status and Molecular Subtypes

**DOI:** 10.1007/s11307-019-01383-w

**Published:** 2019-06-17

**Authors:** Doris Leithner, Blanca Bernard-Davila, Danny F. Martinez, Joao V. Horvat, Maxine S. Jochelson, Maria Adele Marino, Daly Avendano, R. Elena Ochoa-Albiztegui, Elizabeth J. Sutton, Elizabeth A. Morris, Sunitha B. Thakur, Katja Pinker

**Affiliations:** 1 Department of Radiology, Breast Imaging Service, Memorial Sloan Kettering Cancer Center, 300 E 66th St, 7th Floor, New York, NY 10065 USA; 2 Department of Diagnostic and Interventional Radiology, University Hospital Frankfurt, Frankfurt, Germany; 3 Department of Biomedical Sciences and Morphologic and Functional Imaging, University of Messina, Messina, Italy; 4 Department of Breast Imaging, Breast Cancer Center TecSalud, ITESM Monterrey, Monterrey, Nuevo Leon Mexico; 5 Department of Medical Physics, Memorial Sloan Kettering Cancer Center, New York, NY USA; 6 Department of Biomedical Imaging and Image-guided Therapy, Molecular and Gender Imaging Service, Medical University of Vienna, Waehringer Guertel 18-20, 1090 Vienna, Austria

**Keywords:** Radiomics, Diffusion-weighted, Magnetic resonance imaging, Breast cancer, Molecular subtypes, Receptors

## Abstract

**Purpose:**

To compare annotation segmentation approaches and to assess the value of radiomics analysis applied to diffusion-weighted imaging (DWI) for evaluation of breast cancer receptor status and molecular subtyping.

**Procedures:**

In this IRB-approved HIPAA-compliant retrospective study, 91 patients with treatment-naïve breast malignancies proven by image-guided breast biopsy, (luminal A, *n* = 49; luminal B, *n* = 8; human epidermal growth factor receptor 2 [HER2]-enriched, *n* = 11; triple negative [TN], *n* = 23) underwent multiparametric magnetic resonance imaging (MRI) of the breast at 3 T with dynamic contrast-enhanced MRI, T2-weighted and DW imaging. Lesions were manually segmented on high b-value DW images and segmentation ROIS were propagated to apparent diffusion coefficient (ADC) maps. In addition in a subgroup (*n* = 79) where lesions were discernable on ADC maps alone, these were also directly segmented there. To derive radiomics signatures, the following features were extracted and analyzed: first-order histogram (HIS), co-occurrence matrix (COM), run-length matrix (RLM), absolute gradient, autoregressive model (ARM), discrete Haar wavelet transform (WAV), and lesion geometry. Fisher, probability of error and average correlation, and mutual information coefficients were used for feature selection. Linear discriminant analysis followed by k-nearest neighbor classification with leave-one-out cross-validation was applied for pairwise differentiation of receptor status and molecular subtyping. Histopathologic results were considered the gold standard.

**Results:**

For lesion that were segmented on DWI and segmentation ROIs were propagated to ADC maps the following classification accuracies > 90% were obtained: luminal B *vs.* HER2-enriched, 94.7 % (based on COM features); luminal B *vs.* others, 92.3 % (COM, HIS); and HER2-enriched *vs.* others, 90.1 % (RLM, COM). For lesions that were segmented directly on ADC maps, better results were achieved yielding the following classification accuracies: luminal B *vs.* HER2-enriched, 100 % (COM, WAV); luminal A *vs.* luminal B, 91.5 % (COM, WAV); and luminal B *vs.* others, 91.1 % (WAV, ARM, COM).

**Conclusions:**

Radiomic signatures from DWI with ADC mapping allows evaluation of breast cancer receptor status and molecular subtyping with high diagnostic accuracy. Better classification accuracies were obtained when breast tumor segmentations could be performed on ADC maps.

## Introduction

With the revelation that breast cancer is a genetic disease, it has become evident that traditional classifications based on tumor histology, size, grade, and receptor status cannot fully capture its characteristics. Gene expression profiling has revealed four main intrinsic molecular subtypes of breast cancer that show substantial differences in phenotypic presentation, prognosis, treatment response, and outcome [1–5]: luminal A, luminal B, human epidermal growth factor receptor 2 (HER2)-enriched, and triple negative (TN) [6–8]. The assessment of molecular subtypes to guide treatment selection is currently performed by gene expression profiling or immunohistochemical surrogates from tissue samples [8–10]. However, this approach can be limited, as biopsy can only capture a small part of a potentially heterogeneous lesion. Additionally, tumor biology may change over time and during treatment due to epithelial–mesenchymal transition [11].

The emerging field of radiomics relies on the extraction of mathematical patterns that are hidden within medical images, some of which the human eye may not be able to assess, let alone quantify; these patterns may potentially predict disease genotypes and enable spatio-longitudinal monitoring of the entire tumor. Previous radiomic studies in breast cancer have primarily focused on features derived from dynamic contrast-enhanced magnetic resonance imaging (DCE-MRI) and their utility for the prediction of molecular subtype [12–15], as well as tumor histology [16], risk of recurrence [17, 18], response to chemotherapy [19], and potential to metastasize [20, 21]. While DCE-MRI is undoubtedly the most sensitive modality for the assessment of breast cancer, complementary techniques such as diffusion-weighted imaging (DWI) that provides additional functional information have been developed to increase its specificity. DWI with apparent diffusion coefficient (ADC) mapping visualizes diffusivity, which indirectly reflects cell density in solid tumors.

Nevertheless, little is known about radiomic signatures derived from DWI for breast cancer characterization: the small number of previous studies of radiomics of DWI primarily evaluated differentiation between benign and malignant lesions [22–24] or solely used histogram features for the differentiation of TN cancers *vs*. other breast cancer subtypes [25]. Moreover, the actual parameters used to perform DWI radiomics are not yet well established, *i.e.,* whether only the actual solid tumor itself should be measured or if the inclusion of edema, necrosis, and inflammation is essential for the differentiation between subgroups [26, 27].

Due to the recent controversy about the safety of gadolinium-based contrast agents, there is considerable interest to develop unenhanced MRI techniques for improved breast cancer diagnosis and characterization [28–30]. Therefore investigation of DWI radiomic signatures for the differentiation of molecular subtypes is of interest and may have manifold applications such as unenhanced assessment of tumor heterogeneity for improved biopsy planning to better guide treatment decisions or the non-invasive tumor monitoring.

We hypothesized that the spatial heterogeneity of tissue diffusivity differs between molecular breast cancer subtypes. Therefore, the aim of this study was to evaluate the diagnostic performance of radiomic features extracted from standardized DWI data, using different approaches of tumor segmentation, for the assessment of breast cancer receptor status and molecular subtypes.

## Material and Methods

This single-institution retrospective study conforms to Health Insurance Portability and Accountability Act guidelines and was approved by the Institutional Review Board with a waiver of written informed consent.

### Patients

A database search was performed for patients who underwent state-of-the-art multiparametric MRI of the breast including DCE-MRI and DWI from January 2011 to January 2013. Inclusion criteria were the following: age ≥ 18 years; biopsy-proven invasive breast cancer; not pregnant or breastfeeding; and lesion size ≥ 1 cm to reduce the influence of partial volume effect on radiomic analysis [31]. One hundred and seventeen consecutive patients matched the inclusion criteria. Of those, twenty-six were excluded due to prior treatment, poor image quality, or histopathology results showing types of cancer other than invasive ductal carcinoma and invasive lobular carcinoma. Exclusion criteria also included personal history or breast cancer and high-risk status. Therefore 91 women were included in the study.

### MR Imaging

All MRI examinations were performed using a 3 Tesla system (Discovery MR750; GE Healthcare, Milwaukee, WI) with the body coil as transmitter and a dedicated 16-channel phased-array breast coil (Sentinelle Vanguard, Toronto, Canada) as a receiver. The state-of-the-art MRI protocol included the following DWI sequence; a 2D, single-shot, dual spin echo-planar DWI sequence (TR 6.000 ms; minimum TE; flip angle 90^o^; acquisition matrix 98 × 98 or 128 × 128; reconstructed matrix 256 × 256; FOV 28–38 cm; slice thickness 4 or 5 mm; NEX 3; slice gap 0–1 mm; fat suppression, enhanced; parallel imaging, ASSET; acquisition time approximately 2 min for 2 b-values 0 and 1000). The details of the full MRI sequence protocol are provided in a different study [32].

### Radiomic Analysis

Feature extraction was performed semi-automatically using the publicly available software package MaZda 4.6 (http://www.eletel.p.lodz.pl/programy/mazda/), which was developed within the COST (European Cooperation in The Field of Scientific and Technical Research) projects B11 and B21 and has been used in previous studies in the field [31, 33]. In the present study, radiomic analysis was performed exclusively using ADC maps—the latter were chosen over high b-value images as they are less prone to some type of artifacts such as dielectric artifacts.

**Fig. 1:**
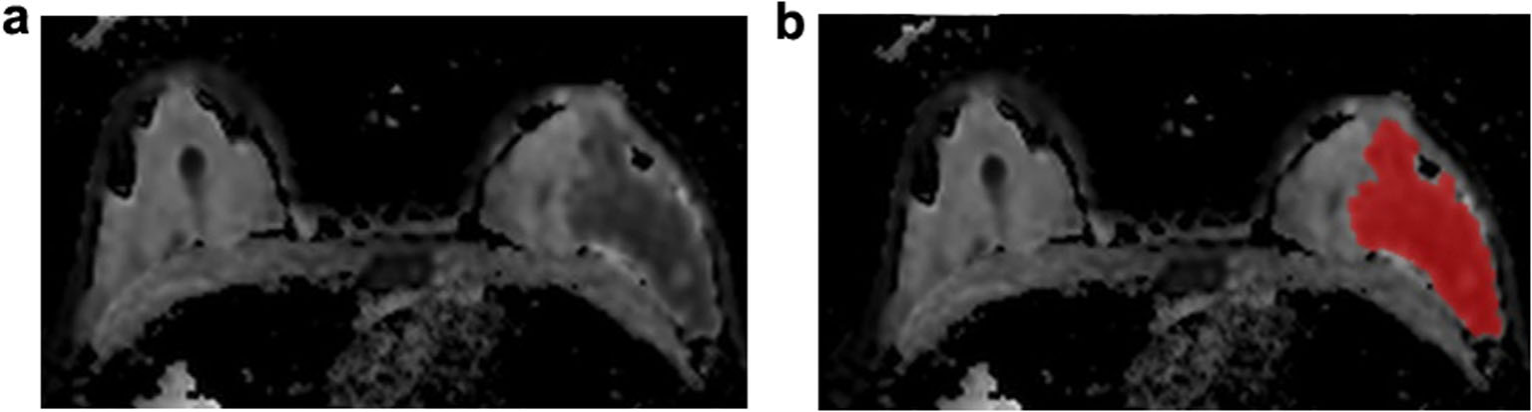
**a** The ADC map for radiomic analysis in a 44-year-old patient with a luminal B invasive ductal carcinoma in the left breast with **b** manual region of interest (ROI) placement.

Two different approaches of tumor segmentation were compared: A single two-dimensional region of interest (ROI): (a) drawn along the visible tumor margins on high b-value DWI and subsequently copied to the corresponding ADC map; and (b) drawn directly along the visible tumor margins on the ADC map. The first approach was performed in all patients, while the second was applied only when an area of decreased ADC values was confidently identifiable on the ADC maps alone without the need for correlation with the high b-value images. Two radiologists (K.P, 13 years of experience; D.L, 4 years of experience) in consensus drew all ROIs on the slice with the largest transaxial lesion diameter on high b-value images or the ADC map (Fig. 1). Adequate distance was kept from the surrounding anatomic structures and biopsy markers. Every tumor was segmented using a freehand ROI, which could be adapted freely in the case of artifacts due to biopsy markers. Artifacts were always excluded from segmentation; even when no artifacts were visible, a distance of at least 2 mm to the marker was kept. DCE-MRI was used to confirm tumor localization in cases that were equivocal on DWI alone.

Gray-level normalization of each ROI was performed using the limitation of dynamics to *μ* ± 3*σ* (*μ*, gray-level mean; *σ*, gray-level standard deviation) to reduce the influence of contrast and brightness variation which might otherwise have an influence on feature quantification [34]. Radiomic analysis included calculation of features derived from first-order histogram (HIS), co-occurrence matrix (COM), run-length matrix (RLM), absolute gradient, autoregressive model (ARM), discrete Haar wavelet transform (WAV), and lesion geometry, as previously described [35–39]. The total time for lesion segmentation and radiomic analysis was approximately 2–3 min per ROI.

### Histopathological Analysis

Tumor samples were analyzed with regard to tumor histology, grade, and immunohistochemical status including estrogen receptor, progesterone receptor, and HER2. Estrogen or progesterone receptor positive tumors (> 1 % staining) were classified as hormone receptor positive. Histological analysis of the surgical specimen was used as reference standard. Molecular subtypes were classified as luminal A for hormone receptor positive and HER2 negative, luminal B for hormone receptor positive and HER2 positive, HER2-enriched for hormone receptor negative and HER2 positive, and TN for hormone receptor and HER2 negative [1, 4, 40]. Fluorescence *in situ* hybridization was used to detect gene amplification in patients with equivocal HER2 status.

### Statistical Analysis

Statistical data analysis was done directly within the MaZda software. Fisher (ratio of between-class to within-class variance), minimization of probability of classification error and average correlation (POE + ACC) and mutual information (MI) coefficients, as previously described [35], were used for radiomic feature selection—each technique provided a set of ten individual features with the highest discriminatory potential. In the next step, the dimensionality of the features sets was reduced using linear discriminant analysis, which generated most discriminatory features (MDFs). Based on these MDFs, *k*-nearest neighbor (*k*-NN) classification (which assigns a point in data space to the class to which its *k*-nearest neighbors belong*)* with leave-one-out cross-validation (LOOCV) was used for pairwise radiomic-based differentiation between molecular subtypes/receptor status [41, 42]. Hence, the model was trained using all patients in the sample (*n*)-1 and validated on the remaining patient. This process was repeated *n* times, with *n* depending on the number of patients in each sample—*e.g.*, for differentiation between luminal A (*i* patients) and luminal B (*j* patients) cancers, *n* = *i* + *j*. This combination of feature selection, reduction and *k*-NN with LOOCV has been used recommended and used in previous studies in the field [31, 33, 35]. The percentage of misclassified data vectors based on the MDFs was used as the main outcome variable, with the true class affiliations, as determined by histopathological analysis (see below) which served as the reference standard. Feature maps were reconstructed to visualize differences in single radiomic features between breast cancer subgroups. These feature maps were used for illustrative purposes only.

## Results

Of the 91 treatment-naïve, biopsy-proven breast cancers, 57 were hormone receptor positive (62.6 %). Forty-nine cancers were classified as luminal A (53.8 %), 8 as luminal B (8.8 %), 11 as HER2-enriched (12.1 %), and 23 as TN (25.3 %). There were 70 mass lesions and 21 non-mass enhancing lesions on DCE-MRI. Mean lesion size was 3.5 ± 2.3 cm (range, 1–16.6 cm). Mean patient age was 48 ± 9.7 years (range, 27–68 years).

Of the 91 lesions, in 79 lesions, areas of decreased ADC values were confidently identifiable on ADC maps alone without the need for correlation with the high b-value images (41 luminal A, 6 luminal B, 10 HER2-enriched, 22 TN). Accordingly, in these 79/91 lesions, tumor segmentation was performed using ROIs constructed on DWI (and then copied to the ADC maps) on the one hand, and using ROIs directly drawn on the ADC maps on the other hand, for comparative analysis. In the remaining 12/91 patients where areas of decreased ADC values were difficult to identify without correlation with high b-value images, tumor segmentation was performed exclusively on high b-value images and then copied to the ADC maps. These twelve lesions included smaller cancers (mean size, 2.7 *vs.* 3.7 cm), non-mass enhancement, and/or very dense breasts.

Radiomics-based classification accuracies ≥ 80 % were considered to be clinically relevant and are listed below:

### Molecular Breast Cancer Subtypes

Best results in terms of accuracies using tumor segmentation directly on the ADC map were achieved for luminal B cancers: luminal A *vs.* luminal B, 91.5 % (POE + ACC/based on COM and WAV features); luminal B *vs.* HER2-enriched, 100 % (Fisher/based on COM and WAV features); luminal B *vs.* TN, 89.3 % (POE + ACC/based on COM features); and luminal B *vs.* all others, 91.1 % (Fisher/based on WAV, ARM, and COM features).

In addition, the separation of HER2-enriched cancers was successful, yielding the following accuracies: HER2-enriched *vs.* luminal A, 80.4 % (Fisher/based on COM features); HER2-enriched *vs.* TN, 81.3 % (POE + ACC/based on COM features); and HER2-enriched *vs.* all others, 81 % (MI/based on COM features).

Likewise, using DWI for ROI delineation, best results in terms of accuracies were achieved for luminal B cancers: luminal A *vs.* luminal B, 89.5 % (MI/based on COM features); luminal B *vs.* HER2-enriched, 94.7 % (MI/entirely based on COM features); and luminal B *vs.* all others, 92.3 % (Fisher/mainly based on COM and HIS features).

The separation of HER2-enriched cancers from other breast cancers was successful, with the following accuracies: HER2-enriched *vs.* luminal A, 83.3 % (Fisher/based on COM and RLM features); and HER2-enriched *vs.* all others, 90.1 % (Fisher/based on RLM and COM features).

In summary, luminal B and HER2-enriched breast cancers show distinct radiomic signatures that enable their separation from other subtypes. Tumor delineation directly on the ADC map yielded higher accuracies than on high b-value images.

### Receptor Status

With regard to hormone receptor status, the accuracy for separating hormone receptor-positive *vs.* HER2-enriched cancers was 84.2 % (MI/based on COM features) in the subgroup with tumor delineation directly on the ADC map. All other accuracies were below 80 %.

All radiomic-based classification accuracies are given in Tables 1 and 2.

**Table 1:** Results of groupwise texture-based cancer classifications using tumor segmentation on the ADC map

Subtypes/receptor status	Luminal A	Luminal B	HER2-enriched	TN	HR positive	HER2 positive	HR negative	HER2 negative	All others
Luminal A	–	*91.5 %* (POE)	*80.4 %* (Fisher)	61.9 % (POE)	–	–	–	–	60.8 % (Fisher)
Luminal B	*91.5 %* (POE)	–	*100 %* (Fisher)	*89.3 %* (POE)	–	–	–	–	*91.1 %* (Fisher)
HER2-enriched	*80.4 %* (Fisher)	*100 %* (Fisher)	–	*81.3 %* (POE)	*84.2 %* (MI)	–	–	–	*81 %* (MI)
TN	61.9 % (POE)	*89.3 %* (POE)	*81.3 %* (POE)	–	73.9 % (Fisher)	76.4 % (MI)	–	–	73.4 % (POE)
HR positive	–	–	*84.2 %* (MI)	73.9 % (Fisher)	–	–	60.8 % (MI)	–	60.8 % (MI)
HER2 positive	–	–	–	76.4 % (MI)	–	–	–	79.8 % (MI)	79.8 % (MI)
HR negative	–	–	–	–	60.8 % (MI)	–	–	–	60.8 % (MI)
HER2 negative	–	–	–	–	–	79.8 % (MI)	–	–	79.8 % (MI)
All others	60.8 % (Fisher)	*91.1 %* (Fisher)	*81 %* (MI)	73.4 % (POE)	60.8 % (MI)	79.8 % (MI)	60.8 % (MI)	79.8 % (MI)	–

Accuracies > 80% are italicized

*HER2* human epidermal growth factor receptor 2, *HR* hormone receptor, *MI* mutual information, *POE* probability of error and average correlation, *TN* triple negative

**Table 2:** Results of groupwise texture-based cancer classifications using tumor segmentation on high b-value DWI and transfer to the ADC map

Subtypes/receptor status	Luminal A	Luminal B	HER2-enriched	TN	HR positive	HER2 positive	HR negative	HER2 negative	All others
Luminal A	–	*89.5 %* (MI)	*83.3 %* (Fisher)	75 % (MI)	–	–	–	–	64.8 % (POE)
Luminal B	*89.5 %* (MI)	–	*94.7 %* (MI)	71 % (Fisher)	–	–	–	–	*92.3 %* (Fisher)
HER2-enriched	*83.3 %* (Fisher)	*94.7 %* (MI)	–	79.4 % (Fisher)	77.9 % (Fisher)	–	–	–	*90.1 %* (Fisher)
TN	75 % (MI)	71 % (Fisher)	79.4 % (Fisher)	–	73.8 % (POE)	66.7 % (Fisher)	–	–	73.6 % (MI)
HR positive	–	–	77.9 % (Fisher)	73.8 % (POE)	–	–	63.7 % (MI)	–	63.7 % (MI)
HER2 positive	–	–	–	66.7 % (Fisher)	–	–	–	73.6 % (Fisher)	73.6 % (Fisher)
HR negative	–	–	–	–	63.7 % (MI)	–	–	–	63.7 % (MI)
HER2 negative	–	–	–	–	–	73.6 % (Fisher)	–	–	73.6 % (Fisher)
All others	64.8 % (POE)	*92.3 %* (Fisher)	*90.1 %* (Fisher)	73.6 % (MI)	63.7 % (MI)	73.6 % (Fisher)	63.7 % (MI)	73.6 % (Fisher)	–

Accuracies > 80% are italicized

*HER2* human epidermal growth factor receptor 2, *HR* hormone receptor, *MI* mutual information, *POE* probability of error and average correlation, *TN* triple negative

## Discussion

In this study, we investigated the diagnostic performance of radiomic signatures derived from DWI for the assessment of breast cancer receptor status and molecular subtypes. We hypothesized that the spatial heterogeneity of tissue diffusivity differs between molecular breast cancer subtypes and could be quantified by radiomic analysis. Our results demonstrate that DWI radiomic features enable the separation between breast cancers of different receptor status and molecular subtypes with high accuracy. Two different approaches of tumor segmentation were explored in this context. Tumor segmentation directly on the ADC map yielded better results in terms of accuracy, suggesting that the solid tumor components strongly contribute to radiomic analysis rather than the inclusion of peritumoral or necrotic tissue. Radiomic signatures derived from DWI may have the potential to enable contrast agent-free spatio-longitudinal monitoring of tumor biology before and during treatment.

One of the main findings of our study is the high accuracy in the radiomic-based discrimination of luminal B and HER2 enriched breast cancers (accuracies, 100 % and 94.7 %). This specific finding might have a direct clinical consequence, as it might prevent the incorrect exclusion of women from hormone therapy when they have heterogeneous tumors. The important information that hormone receptors are present within the tumor might get lost, as biopsy can only capture a snapshot of a potentially heterogeneous tumor, and after neoadjuvant chemotherapy, no tumor cells might be left.

In the first study that investigated the utility of DWI radiomic signatures, Xie *et al*. investigated DWI and DCE-MRI histogram features for the differentiation of TN from other molecular subtypes with AUCs up to 0.763 [25]. Histogram features do not provide textural information in terms of spatial relationships between the signal intensities of pixels/voxels across a region or volume of interest [35]. This may explain why in this first study that investigated the utility of DWI radiomic signatures, true radiomic feature groups (*e.g.*, COM) performed better.

Other studies have investigated radiomic signatures derived from DWI for the differentiation of benign from malignant breast lesions. Bickelhaupt *et al*. recently evaluated DWI radiomic signatures for the separation between benign and malignant breast lesions [22]. Although DWI radiomics yielded better results than ADC mean alone, a highly experienced breast radiologist using DCE-MRI could not be outperformed. Another study by the same group investigated a radiomic model extracted from kurtosis DWI for lesion characterization [23]. The model reduced false positives from 66 to 20 cases at a predefined sensitivity of greater than 98 %, significantly improving specificity compared with median ADC and apparent kurtosis coefficient alone. It has to be noted that in the aforementioned study, there was heterogeneity in terms of hard- and software; however, voxel size for DWI was kept constant, which might have influenced results. Parekh, *et al*. attempted to separate benign from malignant breast lesions using COM features derived from feature maps of DWI, DCE-MRI, T1-weighted, and T2-weighted imaging [24]. The authors found cellular heterogeneity, evaluated using entropy on the ADC map, to be significantly different between benign and malignant lesions.

In the aforementioned studies, different approaches to derive and investigate DWI radiomic signatures were used which limits generalizability of results. It stands to reason that a prerequisite for the widespread application of radiomics in the future will be the rigorous standardization in terms of data acquisition and analysis across institutions and vendors. An alternative would be the use of deep learning neural networks, *i.e.*, with very large heterogeneous datasets in which the classifier has sufficient training data to learn what the real biological information is. In this respect, investigation of how tumors should ideally be segmented is of interest as it remains unclear whether the ROI should include solid tumor components only or additionally cover cystic, hemorrhagic, necrotic, or inflammated regions [43]. Liu, *et al*. found 95 radiomic features in cervical cancer to be insensitive to ROI variation among T2-weighted images, ADC maps of b800 and b1000, with ADC b1000 features yielding a lower misclassification error [27]. In the present study, only b1000 ADC maps were chosen for radiomic analysis. Recently, Wang, *et al*. compared whole tumor, solid portion, and peritumoral edema ROIs for the differentiation between low-grade and high-grade gliomas [26]. They found that tumor inhomogeneity parameters performed better than the ones derived from solid areas only (*P* = 0.048); nevertheless, only HIS and COM features were calculated. In our study, tumor segmentation directly on ADC maps performed better for the separation of molecular breast cancer subtypes than segmentation and ROI transfer from high b-value DWI, which naturally included more non-solid areas surrounding the lesion. The ADC map represents areas of tumor with actual hindered diffusivity and thus might allow a more accurate assessment of the lesion than high b-value DWI, where T2 shine through might occur. Our results suggest that solid tumor components truly contribute to radiomic analysis; however, further studies with three-dimensional evaluation of DWI radiomics in larger patient cohorts are warranted to confirm our findings.

**Fig. 2:**
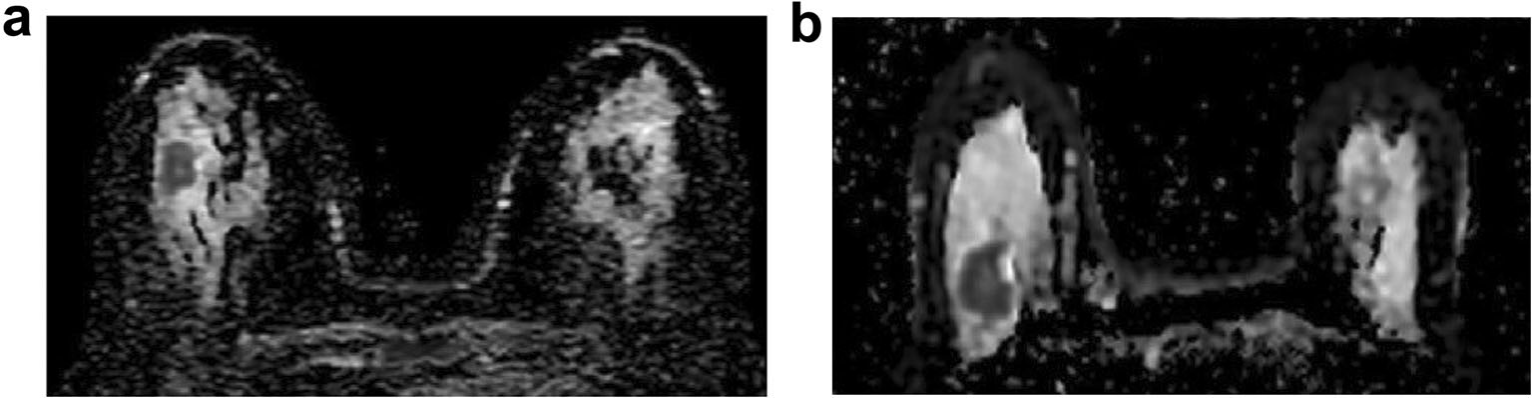
**a** ADC map of a 49-year-old patient with a luminal A cancer in the right breast. **b** ADC map of a 67-year-old patient with a luminal B cancer in the right breast. In our patient collective, radiomic signatures derived from DWI differentiated luminal A from luminal B cancers with an accuracy of 91.5 % when tumor segmentation was performed on the ADC map (89.5 % when segmented on high b-value DWI and copied to the ADC map). (DWI, diffusion-weighted imaging; ADC, apparent diffusion coefficient).

While DWI radiomic features have not been used clinically in this context yet, many previous studies have investigated DCE-MRI radiomic signatures for the separation of breast cancers of different molecular subtypes, although with mixed results. In our study, a multitude of radiomic features from different feature categories was derived from DWI to capture different aspects of image texture, which might have contributed to the excellent results. The majority of DCE-MRI radiomic studies for the separation of molecular subtypes, however, chose to rely solely on a small, typically COM-based subgroup of radiomic features [14, 16, 44, 45]. Sutton, *et al*. demonstrated accuracies of up to 89.2 % for the differentiation between molecular subtypes using a combination of pathology data and COM features [14]. Meanwhile, Holli-Helenius achieved AUC values of 0.83–0.88 for the separation of luminal A and B cancers in a small patient collective (*n* = 27) using COM-features alone [15]. These results are in good agreement with those in our own study (accuracies, 91.5 % and 89.5 %); however, geometric features for shape description performed better than COM features in our study (Fig. 2). In contrast to prior DCE-MRI radiomic studies that show a high level of heterogeneity of scanners and sequence protocols and were only partly successful [4, 12, 13, 16], the acquisition parameters in the present study were strictly homogeneous, which might have had a positive impact on our results. Although DCE-MRI is the most sensitive method for the detection of breast cancer [46, 47], the evaluation of DWI radiomic signatures is of special interest, as they might allow for contrast agent-free tumor biology assessment in times of concern surrounding the safety of gadolinium-containing contrast agents [28–30].

We acknowledge the limitations of our study. Due to the small number of patients in some subgroups, a further division of our population into a training and validation dataset was not considered appropriate. Instead, we decided to apply a *k*-nn classifier with leave-one-out cross-validation, an established method that has been used in multiple studies in the field [31, 48, 49]. Our best results in terms of accuracies were achieved for luminal B and HER2-enriched cancers, a finding that may in part be attributed to the small number of patients in these groups (*n* = 11 and 8, respectively). The mean size of breast cancers included in this study was relatively large, which could explain the high number of triple-negative cancers; at the same time, our results might not be generalizable to smaller lesions in which only few voxel data are available for analysis. Breast cancers were delineated manually, which might introduce a certain level of observer-dependency, and may not be feasible in analyzing large datasets. Furthermore, lesions were segmented two-dimensionally and on the slice with the largest lesion diameter only. Larger prospective studies and multicenter trials with an equal distribution of molecular subtypes are needed to evaluate the robustness of DWI radiomics for the assessment of breast cancer molecular subtypes.

## Conclusion

In conclusion, our preliminary data indicate a possible potential that DWI radiomic signatures enable the assessment of molecular breast cancer subtypes and receptor status with high accuracy. DWI radiomic analysis showed higher accuracies when tumor segmentation was performed directly on ADC maps, indicating that solid tumor areas are essential to radiomic analysis. Although DWI radiomic signatures are unlikely to replace formal genetic testing, with additional validation, they might have the potential to provide a noninvasive, contrast-agent-free method to assess tumor biology before and during treatment.
